# Fully inkjet-printed two-dimensional material field-effect heterojunctions for wearable and textile electronics

**DOI:** 10.1038/s41467-017-01210-2

**Published:** 2017-10-31

**Authors:** Tian Carey, Stefania Cacovich, Giorgio Divitini, Jiesheng Ren, Aida Mansouri, Jong M. Kim, Chaoxia Wang, Caterina Ducati, Roman Sordan, Felice Torrisi

**Affiliations:** 1 Cambridge Graphene Centre, University of Cambridge, 9 JJ Thomson Avenue, Cambridge, CB3 0FA UK; 2 Department of Materials Science and Metallurgy, University of Cambridge, 27 Charles Babbage road, Cambridge, CB3 0FS UK; 3 Key Laboratory of Eco-Textile, Ministry of Education, School of Textiles and Clothing, Jiangnan University, 214122 Wuxi, China; 4 L-NESS, Department of Physics, Politecnico di Milano, Via Anzani 42, 22100 Como, Italy

## Abstract

Fully printed wearable electronics based on two-dimensional (2D) material heterojunction structures also known as heterostructures, such as field-effect transistors, require robust and reproducible printed multi-layer stacks consisting of active channel, dielectric and conductive contact layers. Solution processing of graphite and other layered materials provides low-cost inks enabling printed electronic devices, for example by inkjet printing. However, the limited quality of the 2D-material inks, the complexity of the layered arrangement, and the lack of a dielectric 2D-material ink able to operate at room temperature, under strain and after several washing cycles has impeded the fabrication of electronic devices on textile with fully printed 2D heterostructures. Here we demonstrate fully inkjet-printed 2D-material active heterostructures with graphene and hexagonal-boron nitride (h-BN) inks, and use them to fabricate all inkjet-printed flexible and washable field-effect transistors on textile, reaching a field-effect mobility of ~91 cm^2^ V^−1^ s^−1^, at low voltage (<5 V). This enables fully inkjet-printed electronic circuits, such as reprogrammable volatile memory cells, complementary inverters and OR logic gates.

## Abstract (web summary)

Heterojunction structures based on 2D materials show promise for wearable and textile electronics. Here, the authors demonstrate fully inkjet-printed hetero junctions of graphene and h-BN as a platform for FET-based smart electronics on wearable and washable textile substrates.

## Introduction

Flexible and textile electronics are rapidly growing research fields. Several applications in these fields have been developed over the past two decades, such as organic light-emitting diodes (OLED), photovoltaic devices and transistors^1, 2^. Textile electronics can primarily be grouped into two categories,electronic fibres (e-fibres) and ‘smart’ textiles. E-fibres involve multi-stacking functional layers on the fibres themselves^3^ or combining functional fibres together to create a device^4^. Smart textiles involve the assembly of components on to the weave of the textile and can be done in several ways either through the assembly of components on substrates which can subsequently be placed on textile^5^ or by fabricating devices on the weave of the textile itself^6^.

Many electronic components, such as thermoelectric power generators, sensors, energy storage, antennas and light-emitting devices, have been printed onto textiles for application in curtain lighting^7^, self-heating seats^8^ or wearable electronics (i.e. the integration of electronics with clothes), for monitoring or security purposes, such as motion sensors^9^, health monitoring^10^ and radio-frequency communication^11^. Inkjet printing is a forefront technique for printed electronics^12^. Inks based on organic polymers and metal oxides can be inkjet-printed enabling large-scale manufacturing of transistors on a wide range of plastic substrates. However, both printed metal oxides and organic polymer inks have limited charge mobility (*μ*) (~ 0.01 – 10 cm^2^ V^−1^ s^−1^), which has restricted their prospects to specific applications such as radio-frequency identification tags and control electronics for active matrix displays^13, 14^. Despite their huge potential, there have been only few attempts to deposit field-effect transistors (FETs) onto textiles, such as by coating organic FETs on polymer fibres to create logic inverters^15^, or spin-coating organic FETs on a textile modified with polyurethane/photo acryl layer, achieving *μ* of 0.05 cm^2^ V^−1 ^s^−1^ and on/off ~ 10^5^, respectively^16^.

The exfoliation of layered materials^17^, such as graphite, has driven a surge in the exploration of graphene and more recently other two-dimensional (2D) materials with unique electrical, mechanical, optical and thermal properties^18^. Atomically thin 2D materials, such as graphene and hexagonal-boron nitride (h-BN) can be arranged into heterostructures, thus creating assemblies with novel properties which are different from those of the individual components^17, 18^. The different combination of conducting, insulating and semiconducting 2D materials allows for a large number of different heterostructures with precisely tailored properties with multiple functionalities and improved performance for novel applications^18^. Layered materials can be exfoliated in solution by liquid phase exfoliation (LPE)^19, 20^ or microfluidization^21^ and developed into single or multi-layer 2D-material inks. Graphene and 2D-material printable inks have already shown great potential in the field of printed electronics enabling devices such as thin film transistors^22^, photodetectors^23^, sensors^9^, supercapacitors^24^, saturable absorbers^25^ and solar cells^26^. Layered structures of 2D-material inks can then be printed in part or as a whole by means of different printing technologies such as inkjet, spray, screen, gravure and flexographic printing^12^. In particular, inkjet printing is an ideal tool to print graphene inks enabling FETs^22, 24^ with mobility of up to 95 cm^2^ V^−1^ s^−1^ on surface-modified Si/SiO_2_ substrate^22^ and MoS_2_ photodetectors on Si/SiO_2_ substrate with photoresponsivity of ~36 μAW^−1^ and photocurrent of ~57 pA^27^. Inkjet-printed flexible graphene/MoS_2_ photodetectors on polyethylene terephthalate (PET) showed photoresponsivity of ~500 nAW^−1^ and photocurrent of ~ 30 nA^23^, and more recently inkjet-printed vertically stacked graphene/WS_2_ photodetector were reported with a photoresponsivity up to ~ 1 mAW^−1^ ^28^. More recently, ref. ^29^ demonstrated current modulation in MoS_2_, WS_2_, MoSe_2_ and WSe_2_-printed thin films using electrochemical gating by liquid electrolyte^29^ rather than by heterostructure-based dielectric gating, thus requiring ultra-high vacuum and low temperature for correct device operation, which limits its application to flexible electronics. Moreover, the use of a spray-coated h-BN layer as a porous separator, and not as a dielectric layer may fail to be considered an all-printed 2D-material active heterostructure.

Several issues also still exist in the optimisation of 2D-material inkjet-printed multi-layer heterostructures, which have impeded the inkjet printing of 2D-material-based dielectrically gated field-effect devices, such as FETs. The main manufacturing issue is that the common solvents (e.g *N*-methyl-2-pyrrolidone—NMP, dimethylformamide—DMF) used for 2D-material-based inks are toxic and when printed the different 2D materials in the stack tend to redisperse at the interface if solvent is present, producing uncontrolled interfaces, thus strongly affecting the uniformity of the multi-layer deposition^30^. In addition, the concentration of 2D materials in these solvents is low (<1 mg ml^−1^)^31^, thus requiring many print passes to obtain functional films^30^. Another limitation reported, especially with the use of high boiling point solvents is the required annealing during printing or in post-processing for solvent removal^32^, which poses severe limitations when printing on plastic or textiles. Similar issues are also present in the case of water-based 2D-material-based inks, where the surfactants/polymers removal requires thermal and/or chemical treatments^33^, which are often not compatible with the substrate.

In this work, we demonstrate washable, flexible graphene/h-BN FETs all inkjet-printed with non-toxic, low boiling point inks on polyester textile, and demonstrate its viability for smart textile and printed integrated circuits working at room temperature and pressure. The devices achieved an average field-effect mobility of *μ*_n_ = 150 ± 18 cm^2^ V^−1^ s^−1^ when printed in a coplanar structure on PET and *μ*_h_ = 91 ± 29 cm^2^ V^−1^ s^−1^ on polyester textile.

## Results

### Ink formulation

In this study we used a drop-on-demand (DoD) inkjet printer. The ink viscosity *η* (mPa s), surface tension *γ* (mN m^−1^), density *ρ* (g cm^−3^) and the nozzle diameter *a* (μm) influence the jetting of individual drops from a nozzle^34^. On the one hand, suitable inkjet printable ink formulations, which are produced by LPE^19, 20^ typically contain surfactants or polymer stabilisation agents, which can act as a source of contamination and may hinder device performance, however they can also positively impact the ink by acting as an adhesion or rheology modifier^21^. On the other hand, high boiling point solvents (>100 °C), such as NMP can suspend 2D materials without stabilisation agents due to a matching of the Hansen solubility parameters^19, 20^. However, they are still far from ideal as they are based on toxic and expensive solvents, which require high annealing temperatures (>100 °C) to remove residual solvent^28^. Low boiling point inks (≤100 °C) are a suitable alternative, due to their fast evaporation at room temperature and have been reported to create stable inks though two solvent formulation where the solvent mixture is tuned to improve the affinity of the solvent to the 2D materials^35^. However, the different evaporation rate of the two solvents can result in rheological instabilities and particle aggregation over time. We select an alternative ink formulation route based on solvent exchange, whereby layered materials can be exfoliated effectively in a high boiling point solvent and subsequently transferred to a low boiling-point solvent, with any desired concentration^36^.

We formulated the graphene and h-BN inks (see ‘Methods’) with the following rheological parameters (*η*, *γ* and *ρ*), *η*_h-BN_ ~ 1.7 mPa s, *γ*_h-BN_ ~ 72 mN m^−1^, *ρ*_h-BN_ ~ 1.01 g cm^−3^, *η*_GR_ ~ 1 mPa s, *γ*_GR_~ 30 mN m^−1^, *ρ*_GR_ ~ 0.82 g cm^−3^, which are consistent with previous reports^19, 20, 22^. The inverse Ohnesorge number (Oh) is used as a figure of merit, *Z* = Oh^−1^ = (*γρa*)^1/2^/*η* and is commonly used to characterize the drop formation, stability and assess the jettability of an ink from the nozzle^34, 37^. An overall range of 2 < *Z* < 24 has been identified as an optimal range for printing^22, 37^. We found *Z* ~ 19.4 and *Z* ~ 22 for the h-BN and graphene inks, respectively, which fall within the optimal *Z* range^22, 37^.

**Fig. 1:**
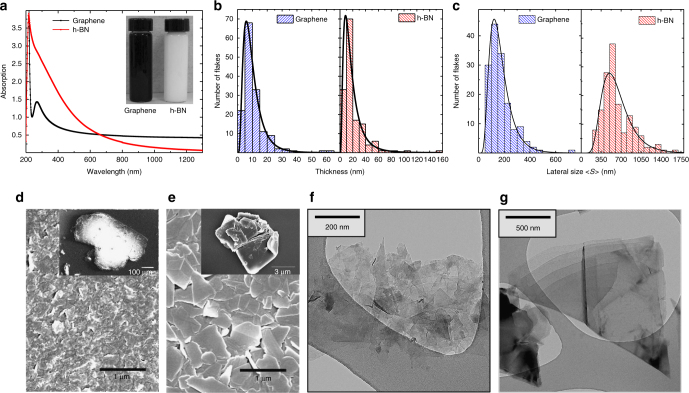
Flakes characterisation. **a** Optical absorption of the graphene and h-BN inks shown in the inset. Atomic force microscopy statistics indicating the thickness (**b**) and the lateral flake size distribution (**c**) for the graphene and h-BN inks. **d** Scanning electron microscopy images of the graphene flakes ~ 200 nm, (inset) starting graphite ~ 400 μm. **e** h-BN flakes ~ 516 nm and (inset) starting bulk h-BN ~ 5 μm. Transmission electron microscopy micrographs of few layer graphene (**f**) and h-BN (**g**) flakes

Optical absorption spectroscopy (Fig. 1a) was used to estimate the flake concentration^19, 20^ *c*, obtaining *c*_h-BN_ ~ 0.44 mg ml^−1^ and *c*_GR_ ~ 0.42 mg ml^−1^ for h-BN (red curve) and graphene (black curve), respectively. The spectra for the graphene ink is mostly featureless due to the linear dispersion of the Dirac electrons^38^, whereas the peak in the UV region is a signature of the Van Hove singularity in the graphene density of states^38^. The spectra for the h-BN ink shows a peak located at 218 nm, which corresponds to the interband transitions in the density of states^39^ revealing an optical bandgap, *E*_g_ of 5.69 eV (see ‘Methods’), which is consistent with previous reports determining the optical band gap for thin h-BN films^40, 41^.

The lateral size $\left( {\left\langle S \right\rangle } \right)$ and thickness (*t*) of the graphene and h-BN flakes was estimated by atomic force microscopy (AFM). Figure 1b shows the statistics of the peak thicknesses extracted from AFM over 150 individual flakes (examples in Supplementary Fig. 1) of graphene and h-BN. The log-normal distribution^42^ is peaked at *t* ~ 6 nm and *t* ~ 9 nm and assuming an approximate 1 nm water layer^29^ and an interlayer distance of 0.34 nm, we calculate that the solutions consist of an average number of layers, *N* ~ 15 and *N* ~ 24 for graphene and h-BN, respectively^43, 44^. The $\left\langle S \right\rangle$ distributions of the flakes contained in each ink (Fig. 1c), defined^42^ as $\left\langle S \right\rangle = \sqrt {xy}$, where *x* and *y* are the length and width of the flake were also investigated to verify that the flake dimensions match with the inkjet requirements for DoD inkjet ($\left\langle S \right\rangle$ ~ 50 times smaller than *a* to avoid nozzle clogging). The lateral size distributions follow a log-normal distribution^42^, which is peaked at ~121 and ~495 nm for graphene and h-BN flakes, respectively. Scanning electron microscopy (SEM) was also used to corroborate the lateral size of the flakes in the inks (Fig. 1d, e). A statistical analysis over 20 flakes for the h-BN and graphene ink (Supplementary Fig. 2a, b), in this case indicates an average $\left\langle S \right\rangle$ of ~520 ± 60 nm and $\left\langle S \right\rangle$ ~ 110 ± 11 nm, respectively, verifying the AFM statistics and the DoD requirements. In each case, we also found that $\left\langle S \right\rangle$ of the bulk material (inset Fig. 1d, e) decreases upon exfoliation by an order of magnitude for the h-BN and more than three orders of magnitude for the graphite, from ~5 and ~400 μm, respectively, indicating the exfoliation of the bulk material into platelets. Figure 1f and g shows transmission electron microscopy (TEM) micrographs of few layer graphene flakes and a terraced h-BN flake and from the graphene and h-BN inks, respectively. The associated TEM statistics (Supplementary Fig. 2c, d) show $\left\langle S \right\rangle$ ~ 760 nm and $\left\langle S \right\rangle$ ~ 120 nm for the h-BN and graphene inks, respectively, which are comparable with the values obtained from AFM and SEM.

### Inkjet-printed graphene/h-BN field-effect heterostructures on PET

**Fig. 2:**
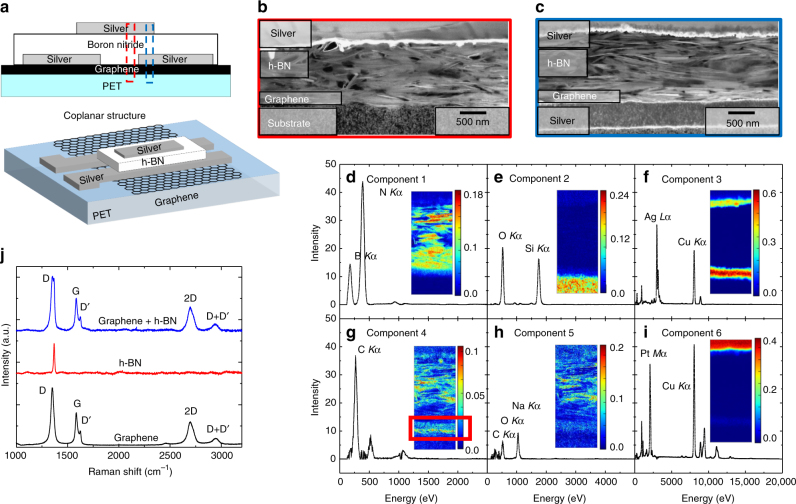
Inkjet-printed coplanar FET on PET. **a** Schematic of a printed coplanar FET heterostructure. HAADF-STEM cross-sectional view of the regions in the red dashed line (**b**) and blue dashed line (**c**). Multivariate analysis of the cross section in the blue region showing non-negative matrix factorization loadings and factors (insets) (**d**–**i**). The red box in **g** indicates the presence of the carbon (i.e. graphene) film. **j** Raman Spectroscopy of the printed coplanar FET heterostructure

We first investigated the dielectric properties of the h-BN ink in a Ag/h-BN/Ag parallel plate capacitor configuration (Supplementary Fig. 3a–e). From the impedance spectra (more details in Supplementary Note 1) of an inkjet-printed capacitor with an area (*A*_c_) of 500 μm^2^ and *t* of 1.2 μm, we estimate a capacitance per unit area is 8.7 nF cm^−2^, which is consistent with the 0.24 to 1.1 nF cm^−2^ range previously reported^45^. Before moving to the textile substrate, we investigated top-gate (coplanar) and bottom-gate (inverted-staggered) FET structures and optimised the inkjet-printed graphene/h-BN heterostructures on a PET substrate (Novele, Novacentrix), which was coated with SiO_2_ nanoparticles (to increase the absorption of residual solvent away from the devices). The coplanar FET heterostructure is shown in the schematics in Fig. 2a. We first inkjet-printed the transistor channel (*t* ~ 100 nm, measured with AFM on Si/SiO_2_) with the graphene ink. Ref. ^22^ determined the percolation threshold of the graphene flakes forming a graphene film at t ~ 25 nm^22^ on a Si/SiO_2_ substrate (*R*_q_ = 0.1 nm, determined by AFM), so for our graphene/h-BN FETs on a rougher PET substrate (*R*_q_ = 68 nm) we print a thicker graphene channel to ensure that graphene flakes fall above the percolation threshold. The source and drain are then printed (*t* ~ 500 nm) using a silver ink (Sigma-Aldrich, 736465) (*Z* ≈ 3). The gate dielectric layer was then inkjet-printed with the h-BN ink forming a film of *t* ~ 1.2 μm and was placed under vacuum overnight at room temperature to remove any bubbles that may be trapped inside the dielectric^46^. Finally, a silver top-gate (*t* ~ 200 nm) electrode was inkjet-printed on the structure and the sample was annealed on a hot plate at 100 °C for 1 h to remove residual solvent. Using the same printing conditions (see 'Methods'), the inverted-staggered FET heterostructures were fabricated by printing a silver gate (*t* ~ 500 nm), then the h-BN dielectric (*t* ~ 1.2 μm) followed by the graphene channel (*t* ~ 100 nm) and finally defining the source and drain electrodes with silver ink (*t* ~ 200 nm), arrays of these devices can be observed in Supplementary Fig. 4a and b.

The morphology and the local chemical composition of the coplanar graphene/h-BN FET devices were investigated using high angle anular dark-field-scanning transmission electron microscopy/energy dispersive X-ray (HAADF-STEM/EDX) analysis. Two lamellae, each ~200 nm wide, were prepared from the coplanar device using focused ion beam (FIB) milling. The cross-sections of the graphene/h-BN FET were extracted from the channel region (red dashed line in Fig. 2a) and from the top/bottom contact region (blue dashed line in Fig. 2a). HAADF-STEM images were acquired on a FEI Tecnai Osiris. The architecture of the channel region is displayed in Fig. 2b, containing the PET substrate, h-BN flakes and the top silver electrode. Figure 2c shows the graphene/h-BN heterostructure sandwiched between drain (bottom) and gate (top) silver electrodes. In both cases, the thickness of the h-BN dielectric is approximately *t* ~ 1.1 μm, consistent with the profilometer analysis for four printing passes discussed in Supplementary Note 1. The h-BN flakes are clearly identifiable and show a preferential alignment along the direction of the channel. The large lateral dimension of our h-BN flakes (peaked at 495 nm) and their orientation ensure significant overlap amongst the flakes, preventing the formation of pin-holes (i.e connections from the top contact to the channel) in the dielectric region^45^. We performed EDX analysis of the lamellae to identify the local chemical composition along the cross section of the heterostructure. The identification of the graphene layer (*t* ~ 100 nm) from the STEM images is very challenging due to its thickness and the coexistence of carbon throughout the layers. In the EDX spectra, the carbon signal is generated both from carboxymethylcellulose sodium salt (CMC), used as stabilisation polymer, and the graphene layer. Therefore, the identification of the carbon presence in the EDX maps is not a good marker for univocally identifying the graphene flakes within the device. Hence, we employed multivariate analysis (see ‘Methods’) to localise EDX signals from compounds, rather than individual elements (Fig. 2d–i) and discriminate between the two carbon-containing phases.

Figure 2d–i shows six spectral profiles associated with the most significant sets of elements (namely—component 1: B and N, component 2: Si and O, component 3: Ag and Cu, component 4: C, component 5: Na, C and O, and component 6: Pt and Cu), containing information on their mutual correlation. The spatial cross-sectional distribution of elements is shown as contour maps in the insets. This representation provides a map of components (e.g. BN or CMC) rather than individual elements (insets Fig. 2d–i). The plot of Fig. 2d shows the EDX energy transitions, where the B *K*_α_ and N *K*_α_ peaks are predominant in component 1 and the inset in Fig. 2d indicates that this component is entirely contained within the dielectric region, as expected, whereas component 2 (exhibiting predominantly O *K*_α_ and Si *K*_α_ peaks) originates from the SiO_2_ nanoparticles coating on the PET substrate (Fig. 2e). Figure 2f shows that component 3 mostly contains Ag *L*_α_ and Cu *K*_α_ lines, which clearly originate from the silver ink (see inset) in the gate and source electrodes and from the TEM grid. In particular, Fig. 2g, h shows components 4 and 5 associated to CMC and graphene, which are carbon-rich compounds. Specifically, component 4 (Fig. 2g) is dominated by the C *K*_α_ peak, and the relative contour map (inset Fig. 2g) highlights two high intensity regions for this component, one at the interface with the silver electrode and a second within the boron nitride flakes. We associate the first region with the presence of graphene (highlighted by a red box, inset Fig. 2g), whereas the second can be linked to the CMC stabilizing the h-BN ink. This is corroborated by evidence that component 5 (containing signal from C, O and Na), typical of CMC, is only present within the dielectric region and not at the interface with the silver electrodes (Fig. 2h, inset), as it should be for the carbon-containing binder. Finally, component 6 is composed of Pt *M*_α_ and Cu *K*_α_, which originate from the platinum deposited to protect the area under investigation (Fig. 2i and inset) and from the TEM grid.

Raman spectroscopy (Renishaw 1000 InVia micro-Raman) is used to monitor the quality of materials used in the heterostructure. Figure 2j plots the spectra of inkjet-printed films of graphene (black curve), h-BN (red curve) and graphene/h-BN heterostructure (blue curve), acquired at 514.5 nm on a Si/SiO_2_ substrate. For the graphene, the G peak located at ~1580 cm^−1^, the D peak located at ~1350 cm^−1^ and the Lorentzian-shaped 2D peak located at ~2695 cm^−1^ indicate that the graphene film comprised of electronically decoupled graphene layers. In pristine graphene inks, the D peak corresponds to the edges of the submicrometer flakes, rather than to the presence of a large amount of disorder within the flakes^22, 47^. This is supported by our graphene film showing a low dispersion of G peak, Disp(G) ≈ 0.01 cm^−1^ nm^−1^, which is lower than what expected for disordered carbon^48^ (see more details in ‘Methods’). In the case of the h-BN film (red curve), we observe a single peak at 1368 cm^−1^ corresponding to the *E*_2g_ phonon vibration mode^49, 50^. Typically for bulk h-BN, this peak is found at 1366 cm^−1^, whereas the upshifting of the peak indicates the successful exfoliation from the bulk material to few layer h-BN^50^. The Raman spectrum of the graphene/h-BN heterostructure (blue curve) shows the fingerprints of graphene flakes and h-BN flakes. The spectrum is in fact the superimposition of that of the graphene film and the h-BN film.

**Fig. 3:**
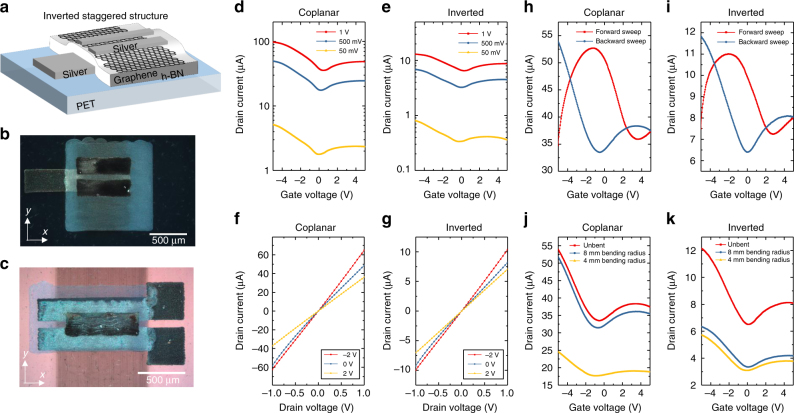
Inkjet-printed inverted-staggered FET on PET. **a** Schematic of a printed inverted-staggered FET heterostructure. Optical microscopy images (dark field) of the **b** inverted-staggered and **c** coplanar FET heterostructures on PET with channel lengths of 50 and 65 μm, respectively. **d**, **e** Transfer characteristics and **f**, **g** linear output characteristics of the FETs as a function of *V*_ds_. **h**, **i** Transfer characteristics of the FETs with observable hysteresis depending on sweep direction at *V*_ds_ = 1 V. **j**, **k** Transfer characteristics of the FETs as a function of bending radius in the *x*-direction at *V*_ds_ = 1 V

We then characterised the output and transfer electrical characteristics of both inverted-staggered (Fig. 3a, b) and coplanar (Fig. 3c) graphene/h-BN FETs on PET. For both structures, the transfer characteristics (shown in Fig. 3d, e) are measured (at room conditions) applying drain-source voltages *V*_ds_=1 V, 500 mV and 50 mV, and the output characteristics (Fig. 3f, g) measured at a gate-source voltage *V*_gs_ = −2, 0, and 2 V indicate that drain current (*I*_d_) increases linearly with *V*_ds_, which is typical of zero-bandgap semiconductors and consistent with the behaviour of graphene FETs^51^. From Fig. 3d, e we observed ambipolar behaviour for both device structures^51^ (*V*_ds_ = 1 V) on all the coplanar and staggered graphene/h-BN-printed devices as is expected for graphene-based FETs^51, 52^. The gate current in all the devices is more than a magnitude lower (<100 nA) than the drain current, indicating that the device is modulating current in the graphene channel. When a forward *V*_gs_ sweep (from negative to positive *V*_gs_) is applied (red curve) in both coplanar and inverted-staggered heterostructures (Fig. 3h, i) the minimum *I*_d_, corresponding to the charge neutrality point (i.e. the Dirac point in graphene), occurs at positive voltage, which means graphene channel was lightly p-doped. We also noticed a hysteretic behaviour for both devices when a forward *V*_gs_ sweep (from more negative to more positive *V*_gs_) is applied (red curve), which causes a shift of the Dirac point of Δ*V* ~ 3 V between forward *V*_gs_ sweep and backward *V*_gs_ sweep (as shown in Fig. 3h, i). This is consistent with ΔV already observed in FETs fabricated by CVD-grown graphene and mechanical cleavage graphene^53^. In our devices, we attribute this effect to capacitive gating created by the oppositely charged ions of residual water in the device, which can enhance the local electrical field at the graphene/h-BN interface and help attract more majority carriers through the metallic contact^53^.

The field-effect mobility of the coplanar and inverted-staggered devices were derived from the slope of the transfer characteristic according to *μ* = (*L*/*WCV*_ds_)/(d*I*_d_ */*d*V*_gs_), where *L* (μm) and *W* (μm) are the channel length and width, respectively, and *C* is the dielectric capacitance^51^. We use *L* ≈ 50 μm, *W* ≈ 580 μm and *L* ≈ 65 μm and *W* ≈ 500 μm for coplanar and inverted-staggered FETs, respectively, and the previously calculated dielectric capacitance per unit area of ~8.7 nF cm^−2^ at *V*_ds_ = 1 V_._ The average field-effect mobility for holes (*μ*_h_) and electrons (*μ*_e_) for the coplanar devices are calculated to be *μ*_h_ ~ 150 ± 18 cm^2^ V^−1^ s^−1^ and *μ*_e_ ~ 78 ± 10 cm^2^ V^−1^ s^−1^, respectively, whereas having an on/off current ratio (defined as the maximum *I*_d_ divided by the minimum *I*_d_) of ∼2.5 ± 0.1. The best device demonstrated a mobility of *μ*_h_ ~ 204 cm^2^ V^−1^ s^−1^ and *μ*_e_ ~ 118 cm^2^ V^−1^ s^−1^. For the inverted-staggered devices, we find an on/off ratio of ∼1.5 ± 0.2, *μ*_h_ ~32 ± 5 cm^2^ V^−1^ s^−1^ and *μ*_e_ ~10 ± 4 cm^2^ V^−1 ^s^−1^, which are one magnitude lower than the coplanar structure field-effect mobility on PET. We attribute this decrease in mobility to the rougher surface of the h-BN layer (root mean squared roughness, *R*_q_ ~68 nm) in contrast to the PET film (*R*_q_ ~15.2 nm), which could affect the stacking quality of the graphene flakes. Such difference between *μ*_h_ and *μ*_e_ corresponds to a preferential hole conduction over electron conduction, which may be due in part to the unintentional doping of the graphene channel^52, 54^. Moreover, a preferential hole conduction has been reported for various graphene FETs on SiO_2_ substrate, including FETs made with graphene from CVD^55^ and mechanical exfoliation^52^. The field-effect mobility of our graphene/h-BN heterostructure FET is higher than that of printed CNT FETs (*μ* ~20 cm^2^ V^−1^ s^−1^, on/off ~10^4^)^56^ and is about 15 times higher than *μ* in the organic (*μ* ~10.5 cm^2^ V^−1^ s^−1^, on/off  ~10^6^)^57^ and metal-oxide transistors (*μ*_e_ ~9 cm^2^ V^−1^ s^−1^, on/off  ~10^7^)^58^, while comparable to that of inkjet-printed graphene FETs (*μ* ~95 cm^2^ V^−1^ s^−1^, on/off ~10)^22^ and reduced graphene oxide FETs (*μ*~210 cm^2^ V^−1^ s^−1^, on/off ~3)^59^. However, the on/off ratio is lower than that of printed organic, metal-oxide and CNT FETs^57, 58^, but consistent with the on/off measured on previously reported FETs from printed graphene^22, 59^. An array of coplanar FETs is shown in Supplementary Fig. 4a and b. The flexibility of the coplanar and inverted-staggered graphene/h-BN FETs was tested as a function of bending radius using metal rods and the resulting transfer characteristics were compared (Fig. 3j, k). For the coplanar FETs, we observed no change in *μ*_h_ at a bending radius of 8 mm (∼1% uniaxial strain along the *x* axis), while *μ*_h_ drops to ∼19 cm^2^ V^−1^ s^−1^ at a smaller bending radius (4 mm, ∼2% uniaxial strain along the *x* axis). For the inverted-staggered FETs, *μ*_h_ drops to ~6 cm^2^ V^−1^ s^−1^ at a bending radius of 8 mm and remains constant until 4 mm bending radius, as shown in Supplementary Fig. 4c. The stability of a printed coplanar graphene/h-BN FETs was also examined over a 2 year period when stored at room temperature and pressure, and we observe that that devices are still operational (Supplementary Fig. 4d). We attribute this behaviour to both the environmental stability of graphene^60^ and the longevity of the h-BN encapsulating properties^61^.

### All inkjet-printed graphene/h-BN field-effect heterostructures on textile

The roughness (*R*_q_) of textiles affects the electrical properties of printed devices. Therefore, we minimised this effect by adopting a planarization layer. Typical planarization layers such as polydimethylsiloxane (PDMS), polyimide, polyurethane or poly(vinyl alcohol) (PVA) decrease the rms roughness and thus improve the device performance^62^. For example, ref. ^63^ used laminated polyurethane (*t* ~ 20–50 μm) on polyester reducing *R*_q_ from 10 μm to < 5 μm^63^, whereas ref. ^64^ used spin-coated polyimide (*t* ~ 500 nm) on polyimide, reducing *R*_q_ from 2.5 to 0.3 nm^64^. Here we employed polyester satin fabric (Wuxi Yihong Textile Co.) as a substrate for our textile graphene/h-BN FETs because it is highly durable and represents about ~80% of the synthetic fibre market. Polyurethane-coated fabric is identified as having the lowest *R*_q_ (~14.8 μm) after one coating layer (*t* ~ 600 nm), as compared to the other coating layers where *R*_q_ ~ 25–34 μm was measured (Supplementary Fig. 5b). We select a ~12 μm thick polyurethane coating layer when fabricating our FET devices on textile as it shows the minimum roughness (*R*_q_ ~1.9 ± 0.5 μm) for the least number of coatings (Supplementary Fig. 5c). Initial attempts to print thin (between 100 and 200 nm thick) graphene channels (from stripes with *L* ~ 2 mm and *W* ~ 1 mm in a coplanar heterostructures, resulted in highly resistive channel (channel resistance, *R*_c_ > 40 MΩ) due to the *R*_q_ of the substrate being orders of magnitude higher the channel thickness, which hinders the development of a coplanar FET structure. Moreover, increasing the thickness of the graphene layer further will inevitably decrease the transistor mobility^22^. On the other hand, the inverted-staggered heterostructure offers a higher resilience to roughness variation of the substrate than the coplanar structure, given that the channel sits on the top of a thick h-BN dielectric layer, which in this case shows *R*_c_ of ~10 kΩ for a 200 nm thick graphene channel. We thus adopt this layout for our graphene/h-BN FET on textile.

**Fig. 4:**
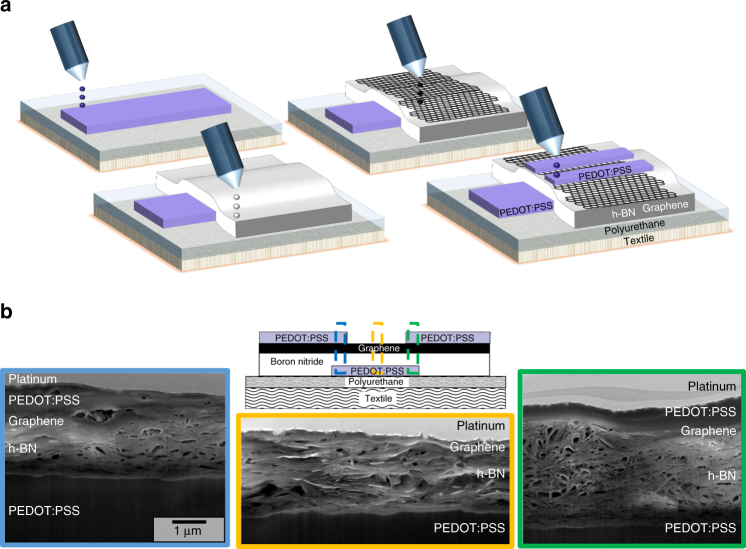
Inkjet-printed FET on textile. **a** Fabrication steps of the inkjet-printed inverted-staggered FET heterostructure on textile. **b** FIB-SEM cross-sectional view of the full FET of the left contact (blue), middle channel (orange), and right contact (green). The scalebar is alike for each figure

**Fig. 5:**
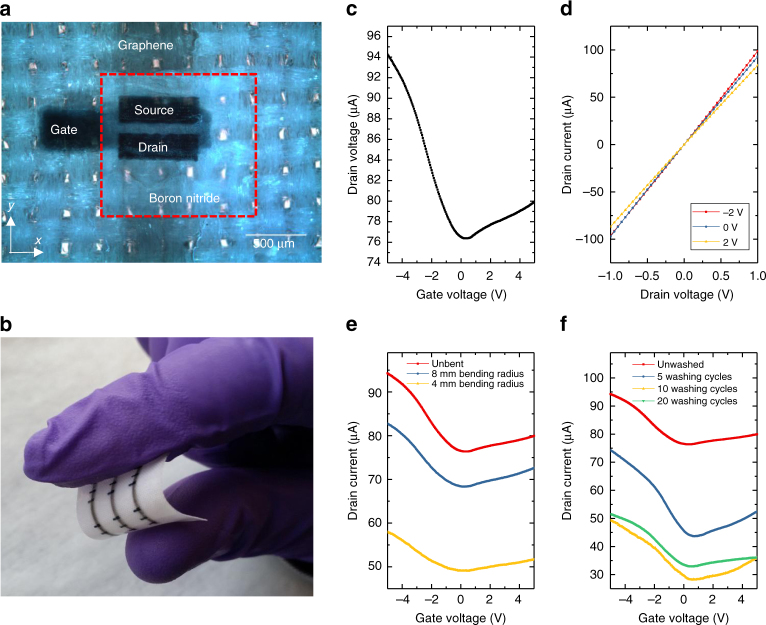
Electrical characterisation of FET on textile. **a** Optical microscopy image of the inverted FET on polyester with a channel length of 80 μm. **b** Image of an array of FETs on textile. **c** Transfer and **d** output characteristics of the textile FET at *V*_ds_ = 1 V. **e** Transfer characteristics of the textile FET as a function of bending radius at *V*_ds_ = 1 V. **f** Transfer characteristics of the textile FET before and after several washing cycles at *V*_ds_ = 1 V

Electronic devices on textile require not only flexibility but the ability to conform with the textile with little or no effect on the electrical and optical performances. Hence, we replace the printed silver electrodes with a highly conformable conductive polymer such as poly(3,4-ethylenedioxythiophene) polystyrene sulphonate (PEDOT:PSS) (Sigma-Aldrich, 739316, 0.8 w/v in H_2_O)^65^. It is worth noting that the PEDOT:PSS ink (*Z* ≈ 30) was successfully jetted from the inkjet nozzles despite being slightly outside the previously found *Z* range (2 < *Z* < 24)^22, 37^. Figure 4a shows each step of the inkjet-printed graphene/h-BN heterostructure on textile. The all inkjet-printed textile FET were fabricated as follows; first we printed a PEDOT:PSS film as the gate electrode (*t* ~ 6.5 μm, determined by profilometry), followed by a h-BN dielectric layer (*t* ∼ 2 μm, corresponding to a capacitance of ∼7 nF cm^−2^) that also reduces residual roughness of the polyurethane planarization layer (*R*_q_ ~ 1.9 μm). Then we printed a ∼200 nm thick graphene channel and finally deposited the PEDOT:PSS source and drain contacts (*t* ∼ 800 nm). We found that the *R*_q_ of the channel decreased further from ~1.9 μm to ~588 nm (as determined by AFM), thanks to the PEDOT:PSS and h-BN bottom layers in the inverted-staggered configuration. Similarly to the FET heterostructures on PET, the samples were annealed at 100 °C for 1 h to remove residual solvent in the devices. Figure 4b shows FIB-SEM cross-sectional views of the FET heterostructure on textile of the regions under the left contact (blue), the middle channel (orange) and the right contact (green), where we identify the graphene layer, h-BN layer and PEDOT:PSS layers in the respective configurations. The layers are continuous and no pin-holes or delaminating areas are visible, even where the substrate varies locally, showing the effectiveness of the planarization and the h-BN layers in minimising the channel roughness. Also in this case, the preferential horizontal orientation of the h-BN flakes in the dielectric layer of the channel region prevents the formation of large vertical gaps, thus avoiding pin-holes. Figure 5a shows an optical microscopy image of the graphene/h-BN FET heterostructure on textile, and Fig. 5b shows an all inkjet-printed array of FET heterostructures on polyester fabric. The geometry of all the graphene/h-BN textile FETs is *L* ≈ 80 μm, *W* ≈ 500 μm and *t* ≈ 200 nm. Figure 5c and d shows, respectively, the transfer characteristic at *V*_ds_ = 1 V and the output characteristic for different gate voltages *V*_gs_ = −2, 0 and 2 V. The average field-effect mobility of the devices is *μ*_h_ ~91 ± 29 cm^2^ V^−1^ s^−1^ and *μ*_e_ ~22 ± 10 cm^2^ V^−1^ s^−1^, respectively, whereas the on/off ratio is ∼1.23 ± 0.3. This behaviour is expected, as *μ* of printed graphene channels increases with thickness until percolation is reached^22^. Our *μ* is two to three magnitudes larger than what has currently been achieved for organic FETs on e-textile fibres (*μ* ~0.01–0.3 cm^2^ V^−1^ s^−1^, on/off ~10^3^)^16, 66^ while reaching one magnitude greater mobility than inverted-staggered FETs spin-coated on polyester textile with a ion gel dielectric/P3HT smoothing layer (*μ* ~7 cm^2^ V^−1^ s^−1^, on/off ~10^5^)^67^. We also printed the same inverted FET with ~100 nm graphene channel thickness, reaching field-effect current modulation (Supplementary Fig. 6a). However, the *R*_c_ ~ 450 kΩ in this case is more than one order of magnitude higher than the inverted FET on PET. By increasing the graphene thickness to ∼200 nm, we reduce *R*_c_ to ~10 kΩ, and in this case we likely reached the bulk conductivity regime, as already shown for inkjet-printed graphene patterns and FET channels^22^ >25 nm thick. It is worth also noting that our device operates at low voltage (<5 V), which is a key requirement for the low-power consumption of textile electronics devices^68^.

Textiles normally undergo naturally occurring tensile strain as well as washing steps^9^ so it is important that electronic devices integrated within these textiles also withstand these steps. Therefore, we investigated the effect of bending (Fig. 5e) and washing cycles (Fig. 5f) on the mobility of our graphene/h-BN FET heterostructures on textile. We find that *μ*_h_ decreases to ~41 cm^2^ V^−1^ s^−1^ at 8 mm bending radius (∼2% uniaxial *x*-axis strain) and to ~27 cm^2^ V^−1^ s^−1^ at 4 mm bending radius (∼4% uniaxial *x*-axis strain, as shown in Supplementary Fig. 4c), with the on/off ratio not significantly changing (Δon/off ∼ 0.05), thus indicating the devices remain operational while under strain (Fig. 5e). Figure 5f shows the transfer characteristics of the inverted-staggered textile FET as a function of washing cycles (see ‘Methods’). To protect the printed FET, a waterproof polyurethane-protective layer (WBM Seam Tapes) was hot pressed (PixMax Swing heat press) around the top and bottom of the devices at 120 °C for 5 s. The graphene/h-BN FET heterostructure was functional up to at least 20 washing cycles without any significant change to the performance of the devices in terms of mobility (Supplementary Fig. 6b) and on/off ratio. The drain current, however decreased from 94 to ~50 μA after 10 washing cycles and maintains this value over 20 washing cycles, most likely due to the increasing channel resistance as a result of the bending the device experienced during washing. The demonstration of washability is important as it prolongs the device lifetime, avoids replacement costs and improves compatibly with current textile industry technologies.

### Electronic circuits based on all-printed 2D-material heterostructures

**Fig. 6:**
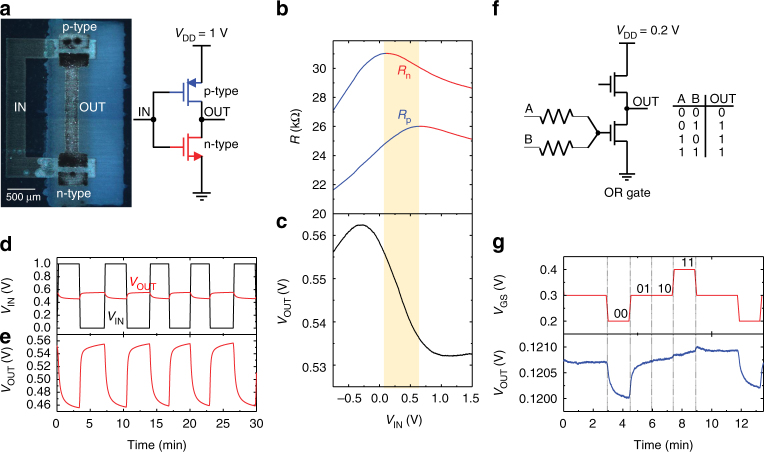
Fully inkjet-printed logic. **a** Optical microscopy image (dark field) of an integrated circuit demonstrating an all inkjet-printed complementary graphene inverter as shown in the schematic. **b** The measured resistances of the top (*R*_p_) and bottom (*R*_n_) GFET as a function of the input voltage. Segments of the curves displaying *n*-type (*p*-type) behaviour are drawn in red (blue). **c** The measured static voltage transfer characteristic of the printed inverter. The inverter is capable of signal inversion between the maximum and minimum of the transfer characteristic. **d**, **e** Digital *V*_IN_ black) and *V*_OUT_(red) waveforms measured on one of the fabricated inverters at *V*_DD_ = 1 V under ambient conditions. **f** Circuit diagram of a multifunctional printed logic gate with two inputs (A and B) and one output (OUT) with truth table of an OR logic gate. **g** Digital waveforms measured in an OR logic gate. The gate voltage *V*_GS_ takes three discrete levels labelled by the values of the corresponding Boolean variables A and B

The versatility of the inkjet-printed FETs allows to arbitrarily design electronic circuits. The goal of an ‘all inkjet-printed’ circuit with 2D-material heterostructures has been a dream pursued for long time^30^. To verify the suitability of our technology for fully printed, flexible electronic circuits with 2D materials, here we demonstrate an all inkjet-printed complementary inverter (Fig. 6a–e), a reprogrammable logic OR gate (Fig. 6f, g), a single transistor memory cell (Supplementary Fig. 7a, b) and p-type and n-type logic inverters (Supplementary Fig. 8a, b) using the all-printed coplanar graphene/h-BN FET heterostructures. Figure 6a shows the optical microscopy image (dark field) of an all inkjet-printed complementary graphene/h-BN inverter, whereas the schematic of the inverter is shown beside it. In Fig. 6b, two printed FETs were connected to a power supply *V*_DD_ = 1 V to create a complementary graphene inverter^69^, with the coplanar graphene/h-BN heterostructures. The Dirac voltages of both FETs were identical at low biases. When *V*_DD_ = 1 V was applied, the larger gate (i.e. input) voltage was required to reach the Dirac point of the top FET with respect to that of the bottom FET^70, 71^. This is illustrated in Fig. 6b in which the resistance maximum of the top FET is shifted to larger input voltages compared to that of the bottom FET. The complementary operation of the FETs was obtained between their Dirac voltages (the area shaded in yellow), where the top FET operated in the p-type and the bottom FET in the n-type regime^69^. Although the strongest signal inversion is obtained in the complementary mode, the inverter is capable of signal inversion in a slightly larger input voltage range^69^, i.e. in the range between the maximum and minimum of the static voltage transfer characteristic shown in Fig. 6c. As this range is approximately equal to the rail-to-rail voltage range (0 to *V*_DD_ = 1 V), the inverter correctly inverted the input digital signal, as shown in Fig. 6c. In Fig. 6d, digital waveform for the input signal (black line) has logical high *V*_DD_ = 1 V and logical low 0 V. The output signal of the inverter (red line) with the input signal is shown in Fig. 6d in the same scale, whereas the output signal is also plotted in Fig. 6e. In principle, the inverter is capable of in/out signal matching because the offsets of the input and output signals are the same (*V*_DD_/2)^72, 73^. The output voltage swing is 10% of the input voltage swing, indicating low voltage gain (*A* = d*V*_OUT_/d*V*_IN_ ≈ 0.1) of the inverter. The reason for the low gain is a very thick (~1 µm) gate insulator, which corresponds to a very large equivalent oxide thickness (EOT) of 557 nm. Nevertheless, the obtained gain is still larger than that of the first exfoliated complementary graphene inverters with EOT = 300 nm^69^.

We also realized an OR logic gate to demonstrate the versatility of the printed graphene/h-BN FET heterostructures. Two printed FETs were connected to a low-voltage power supply *V*_DD_ = 0.2 V as shown in Fig. 6f. We left the gate of the top FET floating (i.e. no bias) and used it as a resistive load (i.e. pull-up resistor) of the bottom FET. Two input logic signals *V*_A_ and *V*_B_ (corresponding to Boolean variables A and B) were connected to the gate of the bottom graphene field-effect transistor (GFET) via two identical resistors. This gives for the gate voltage of the bottom GFET *V*_GS_ = (*V*_A_ + *V*_B_)/2^74, 75^. As the input logic signals had a low *V*_L_ (Boolean 0) or high *V*_H_ (Boolean 1) voltage level, the gate voltage had three discrete levels: low *V*_L_ (both inputs 0), high *V*_H_ (both inputs 1), and half way between (*V*_L_ + *V*_H_)/2 (inputs different). Depending on the position of the gate voltage levels with respect to the Dirac voltage, different logic gates can be obtained^74^. Figure 6g shows the input and output voltage signals in an OR gate. The OR logic function was obtained by choosing the input levels such that the Dirac voltage of the bottom GFET is exactly half way between the mid and high gate level^74^. The low output voltage swing originates from the bottom FET operating very close to the Dirac point due to the small difference between the high (*V*_H_ = 0.4 V) and low (*V*_L_ = *V*_H_ − *V*_DD_ = 0.2 V) level and from the thick gate insulator in the GFETs.

These results demonstrate the robustness of our technology based on all inkjet-printed 2D-material heterostructures to fabricate flexible and integrated electronic circuits, such as memories and logic gates exhibiting the same functionality as the some of the first circuits fabricated by standard lithographic methods with heterostructures of 2D materials ^69^.

## Discussion

We demonstrated fully inkjet-printed dielectrically gated field-effect transistors based on graphene/h-BN heterostructures for flexible and textile electronic circuits. The graphene/h-BN heterostructure FETs on polyester textile show *µ*_h_ and *µ*_e_ up to 91 cm^2^ V^−1^ s^−1^ and 22 cm^2^ V^−1^ s^−1^ in ambient conditions, withstand up to ∼4% strain and are washable for up to at least 20 cycles. The viability of the printed graphene/h-BN heterostructures FETs for electronic circuits was also demonstrated with fully printed complementary inverters, logic gates and rewriteable volatile memory.

## Methods

### Ink preparation

The graphene ink was prepared by dispersing graphite flakes (10 mg ml^−1^, Sigma-Aldrich No. 332461, <100 μm size) and ultrasonicating (Fisherbrand FB15069, Max power 800 W) for 9 h in NMP^19^, providing surfactant/polymer-free exfoliation of graphite. The graphene ink in NMP was then solvent exchanged to ethanol so that we facilitate solvent removal at ambient temperature during printing, thus improving the uniformity of the multi-layer deposition^25^. The h-BN ink was prepared by mixing h-BN powder (10 mg ml^−1^, Goodfellows B516011, <10 μm size) with deionized water and carboxymethylcellulose sodium salt (CMC, average molecular weight *M*_W_ = 700,000, Aldrich No. 419338) (3 mg ml^−1^), a biocompatible and biodegradable stabilisation agent and rheology modifier^21^. The h-BN/CMC mixture was then processed with a shear fluid processor, (i.e. a microfluidizer, M-110P, Microfluidics International Corporation, Westwood, MA, USA) with a Z-type geometry interaction chamber with microchannels ∼87 µm wide for 50 cycles, at 207 MPa system pressure and at room temperature (20 °C)^21^. We used the microfluidic process to disperse and exfoliate h-BN while the high shear rate generated ($\dot \gamma$ ∼ 9.2 × 10^7^ s^−1^) helped to achieve high concentration dispersions^21^. The h-BN and graphene dispersions were then ultracentrifuged (Sorvall WX100 mounting a TH-641 swinging bucket rotor) at 3 k rpm (20 min) and 10 k rpm (1 h), respectively, to remove thick flakes, which would clog the printer nozzles. Subsequently, the supernatant (i.e. top 70%) was decanted for further characterisation.

### Solvent exchange

First (~20 ml) of graphene/NMP ink is passed through a PTFE membrane (Merck Millipore, 0.1 μm). The process is hastened with the use of Büchner flask which is attached to a vacuum pump. The membrane is then placed into 5 ml of ethanol and bath sonicated (Fisherbrand FB15069, Max power 800 W) for 10 min to redisperse the flakes into the ethanol.

### Ink characterisation

The surface tension is measured using the pendent drop method (First Ten Angstroms FTA1000B). The shape of the drop suspended from a needle results from the relationship between the surface tension and gravity. The surface tension is then calculated from the shadow image of a pendant drop using drop shape analysis. A parallel plate rotational rheometer (DHR rheometer TA instruments) is used to evaluate the viscosity as a function of shear rate, the infinite-rate viscosity for each ink is found. Ink density is evaluated from a (Sartorius ME5) microbalance where the density is *ρ* = *m*/*V*, *m* is the mass and *V* the volume.

### Optical absorption spectroscopy

The flake concentration in the graphene and h-BN inks are obtained via the Beer-Lambert law, which correlates the absorbance *A* = *αcl*, with the beam path length *l* (m) the concentration *c* (g l^−1^) and the absorption coefficient *α* (l g^−1^ m^−1^). The h-BN and graphene inks were diluted 1:20 with water/CMC and ethanol, respectively. Absorption coefficients of *α*_h-BN_ ~ 2350 l g^−1^ m^−1^ for the h-BN ink^76^ and *α*_GR_ ~ 2460 l g^−1^ m^−1^ for the graphene ink^19^ at 660 nm were utilised. The optical bandgap of the h-BN ink is obtained using *E*_g_ defined by *hc/λ*_g_, where *h* is the Planck constant, *c* is the speed of light in vacuum and *λ*_g_ is the resonance wavelength of the peak.

### Raman spectroscopy

Films of each ink and a graphene/h-BN heterostructure are inkjet-printed on Si/SiO_2_ substrate and the Raman spectra are acquired with a Renishaw 1000 InVia micro-Raman spectrometer at 457, 514.5, and 633 nm and a 20× objective, with an incident power of below ∼1 mW to avoid possible thermal damage. The system is within 1.5 cm^−1^ spectral resolution at 514 nm. The G peak dispersion is defined as Disp(G) = ΔPos(G)/Δ*λ*_L_, where *λ*_L_ is the laser excitation wavelength. In disordered carbon, the G peak position, Pos(G), increases as the excitation wavelength *λ*_L_ decreases from the IR to UV^77^. Therefore, the dispersion of the G peak, increases with disorder and allows one to discriminate between disorder localised at the edges or in the bulk of the samples^47, 77^.

### Scanning electron microscopy

Scanning electron microscopy images were acquired with a high resolution Magellan 400L SEM. The field emission gun was operated at an accelerating voltage of 5 kV and gun current of 6.3 pA. Images were obtained in secondary electron detection mode using an immersion lens and TLD detector. A 3–5 nm platnium coating was sputtered (Emitech sputter coater) on to the surface of the h-BN flakes to reduce the build-up of electrons.

### Transmission electron microscopy

Drops of boron nitride and graphene inks were dispensed on holey carbon TEM grids and then analysed in a FEI Osiris operating in bright field mode at 80 kV. A FEI Helios dual beam Focus Ion Beam/Field Emission Gun—Scanning Electron Microscope (FIB/FEG-SEM) was employed to prepare lamellae for STEM imaging and analysis. The same microscope was used for slice and view imaging. EDX data were acquired using a FEI Osiris TEM equipped with a high-brightness Schottky X-FEG gun and a Bruker Super-X EDX system composed of four silicon drift detectors, each ~30 mm^2^ in area and arranged symmetrically around the optical axis to achieve a collection solid angle of 0.9 sr. The windowless design of the detector allows qualitative mapping of light elements. Spectrum images were acquired with an acceleration voltage of 80 kV, a spatial sampling of 10 nm pixel^−1^ and 100 ms pixel^−1^ dwell time. We employed multivariate analysis to localise EDX signals from compounds by applying a statistical analysis algorithm (non-negative matrix factorisation—NMF—in this case)^78^ to the EDX dataset, comprising several thousand spectra, and identifying correlations between spectral features. Data were acquired with Tecnai Imaging and Analysis (TIA) and analysed within Hyperspy^79^, an open source analysis tool-kit where statistical analysis algorithms such as NMF, are implemented.

### Atomic force microscopy

A Bruker Dimension Icon working in peakforce mode was used. From the centrifuged graphene and h-BN dispersions, samples were collected and after 10 times dilution they were drop casted onto pre-cleaned (with acetone and isopropanol) Si/SiO_2_ substrates wafer substrates. For the graphene and h-BN inks, 150 flakes were counted to determine the statistics for the lateral size and thickness. For the rms, roughness measurement areas of 50 μm^2^ were scanned.

### Electrical characterisation

All devices were measured with a probe station (Summit 12000, Cascade Microtech) that is connected to a semiconductor device analyser (Agilent Technologies, B1500A) to obtain I-V curves. We use a sweeping time of 0.59 V s^−1^ in all devices.

### Inkjet printing

We used a DoD ink-jet printer (Fujifilm Dimatix DMP-2800) equipped with a 21 µm diameter nozzle (Fujifilm DMC-11610) where the volume of individual droplets from this nozzle is ~10 pl. The platen temperature was kept as 20 °C throughout printing of heterostructures. Each ink was printed at a maximum jetting frequency of 2 kHz. During droplet ejection a primary drop may be followed by secondary (satellite) droplets which need to be avoided during printing^80^. In addition, nozzle clogging can be an issue unless the particles are ~1/50 or less the nozzle diameter^22^. When inkjet printing, the ejected drop contacts the substrate and spreads according to Young’s equation, *γ*_SV_ − *γ*_SL_ − *γ*_LV_ cos *θ*_c_ = 0 (where *γ*_SV_ is the solid–vapour surface energy, *γ*_SL_ the solid–liquid interfacial tension and *γ*_LV_ the liquid–vapour surface tension). The *γ*_LV_ of the graphene ink (30 mN m^−1^) and PEDOT:PSS ink (44 mN m^−1^) is minimised so that the droplets will coalesce even on low *γ*_SV_ surfaces (such as polyurethane) allowing uniform printed lines. Furthermore, similarly to CNTs^81^ low boiling point solvents (≤100 °C) are used to disperse the graphene, h-BN and PEDOT:PSS inks, which evaporate quickly minimising both the transport of particulates (which causes ‘coffee ring’) and the re-dispersion of material at the heterostructure interfaces, thus improving the morphological uniformity of the layered arrangement. The drop then dries through solvent evaporation and the resulting thickness will depend on the number of droplets delivered per unit area (controlled by the interdrop spacing, i.e. the centre - to - centre distance between two adjacent deposited droplets), the drop volume and the concentration of material in the ink.

### Washability test

For each washing test, the sample was washed by immersion in a sealed beaker of 100 ml deionized water containing 2 mg ml^−1^ sodium carbonate and 5 mg ml^−1^ soap at 50 °C, and tumble washed for 30 min according to industry standards^9^.

### Data availability

The data that support the findings of this study are available from the corresponding author upon request.

## Electronic supplementary material

Supplementary Information
