# Supplementary material for: Fully inkjet-printed two-dimensional material field-effect heterojunctions for wearable and textile electronics
Source: Nat Commun. 2017 Oct 31;8:1202. doi: 10.1038/s41467-017-01210-2 (PMC5663939; doi:10.1038/s41467-017-01210-2)
Supplement: Supplementary file 1 — Supplementary Information [file 41467_2017_1210_MOESM1_ESM.pdf]

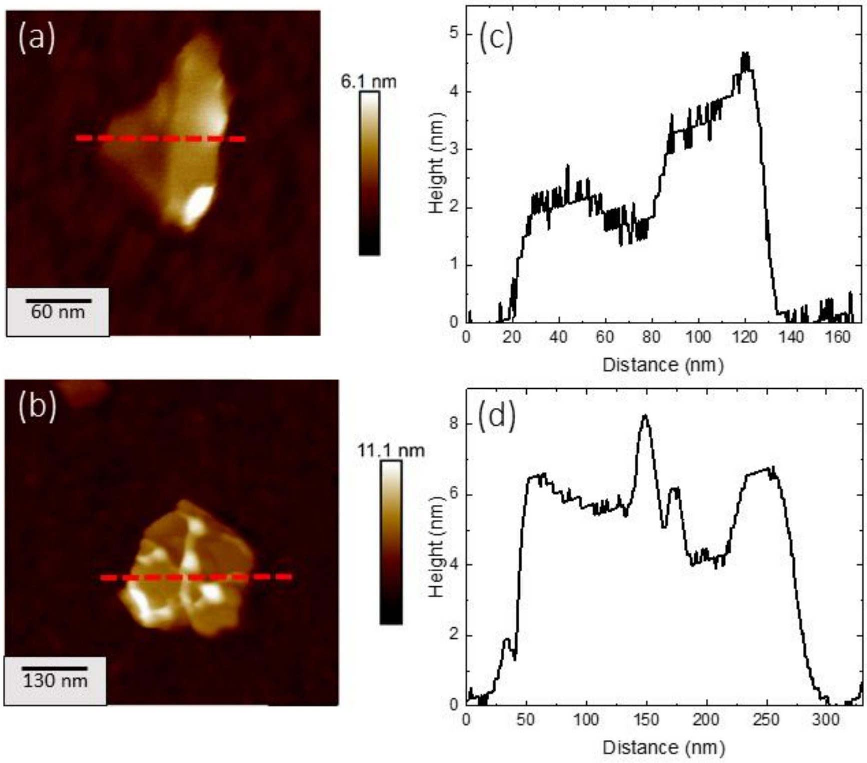

Supplementary Figure 1: Graphene and h-BN flake micrographs. Atomic force microscopy images of typical flakes produced from LPE graphene ink (a) and microfludised h-BN ink (b). Corresponding cross section profiles of h-BN ~300nm (c) and graphene ~100nm (d) flakes.

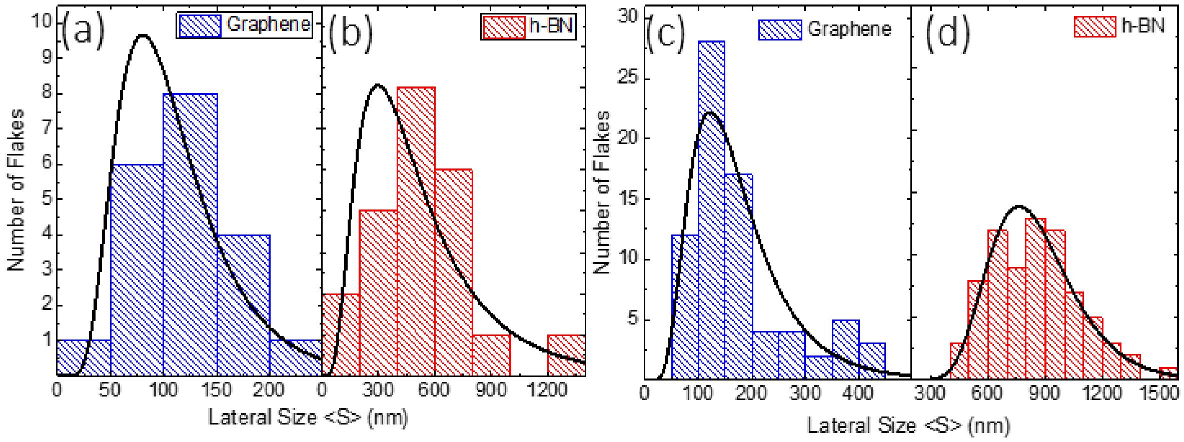

Supplementary Figure 2: Graphene and h-BN flakes statistics. SEM statistics of the Graphene (a) and h-BN flakes (b). TEM statistics indicating the lateral size distribution of the graphene (c) and h-BN flakes (d).

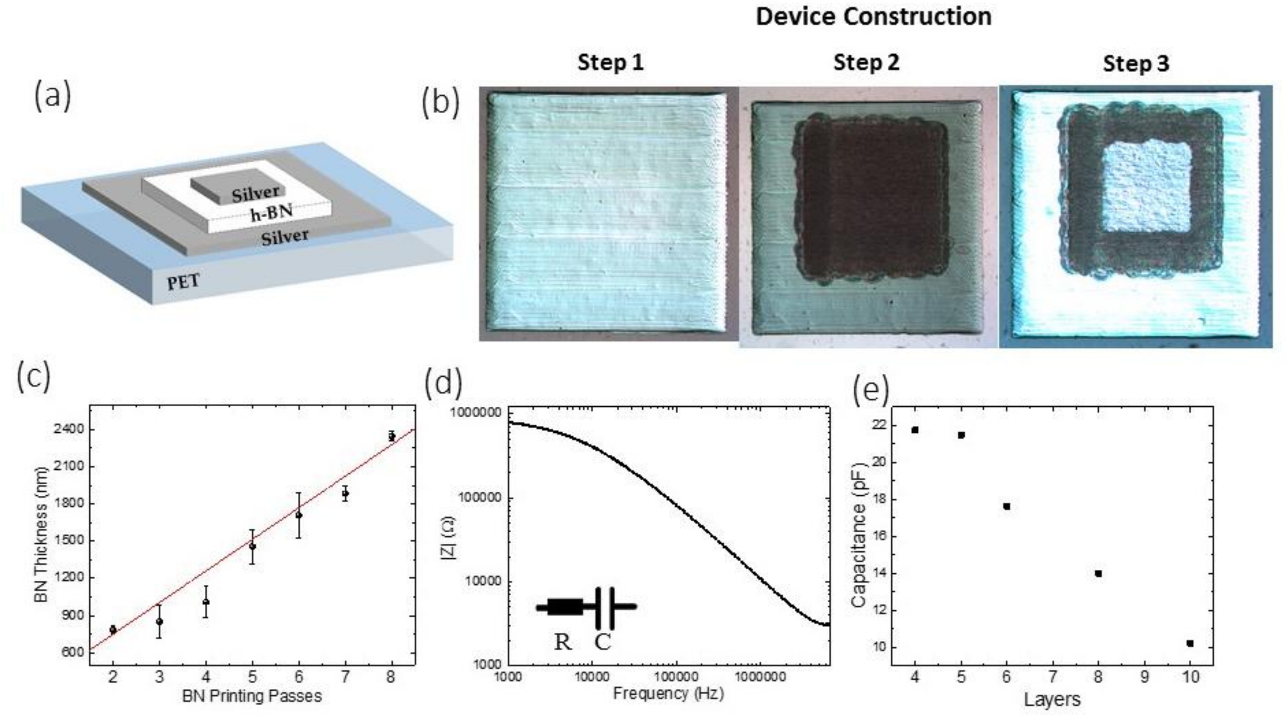

Supplementary Figure 3: Inkjet printed capacitors on PET (a) 3D model of capacitor heterostructure (b) Optical microscopy of each fabrication step for capacitors which have been fully inkjet printed. (c) Stylus profilometry of the h-BN thickness as a function of printing passes. Error bars are calculated using standard deviation of mean. (d) Typical impedance spectra for each capacitor obtained which follows a R-C equivalent circuit model. (e) Capacitance variation with number of printed layers.

Supplementary Note 1: Inkjet Printed h-BN capacitors

We investigate the dielectric properties of the h-BN ink in a Ag/h-BN/Ag parallel plate capacitor configuration (Supplementary figure 3a). The capacitors are fabricated by inkjet-printing silver ink (Sigma Aldrich, 736465) ($Z \approx 3$) and h-BN ink layer-by-layer (Supplementary figure S3b). A profilometer (DektakXT, Bruker) was used to determine the thickness (t) of each printed h-BN film as a function of the number of printing passes (Supplementary figure S3c), where a single printing pass results in t~300nm. To characterise the properties of the capacitors, impedance spectra (Agilent 4294A Precision Impedance Analyzer) were measured for each capacitor with varying h-BN film thickness (from ~1.2μm to ~1.8μm). A typical bode plot of the amplitude ($|Z|$) as a function of frequency are shown in supplementary figure 3d for a capacitor with h-BN film of thickness, t ~1.2μm and area, A_c = 500μm^2 and presents a typical behaviour of a series R-C equivalent circuit where the impedance amplitude is determined by $|Z| = \sqrt{(R_{ser})^2 + (\omega C_{ser})^{-2}}$ [1]. The capacitance (C_{ser}) is found to decrease

with h-BN thickness (Supplementary Figure 3d) as expected through the equation for a parallel plate capacitor $C = \varepsilon_r\varepsilon_0 A_c/t$, where ε_r is the relative permittivity and ε_0 is the vacuum permittivity. For an inkjet-printed capacitor ($A_c = 500\mu m^2$, t~1.2μm) the capacitance per unit area is 8.7 nF/cm^2 (R_{ser} ~11kΩ, C_{ser} ~22 pF) which is consistent with the 0.24 to 1.1nF/cm^2 range previously reported[1] with a graphene/h-BN capacitor deposited by inkjet printing (graphene) and spray coating (h-BN) techniques. In our case, capacitors with dielectric having t < 1 μm were found to short circuit the Ag electrodes. Hence we select t ~1.2μm for our inkjet printed h-BN dielectric layer.

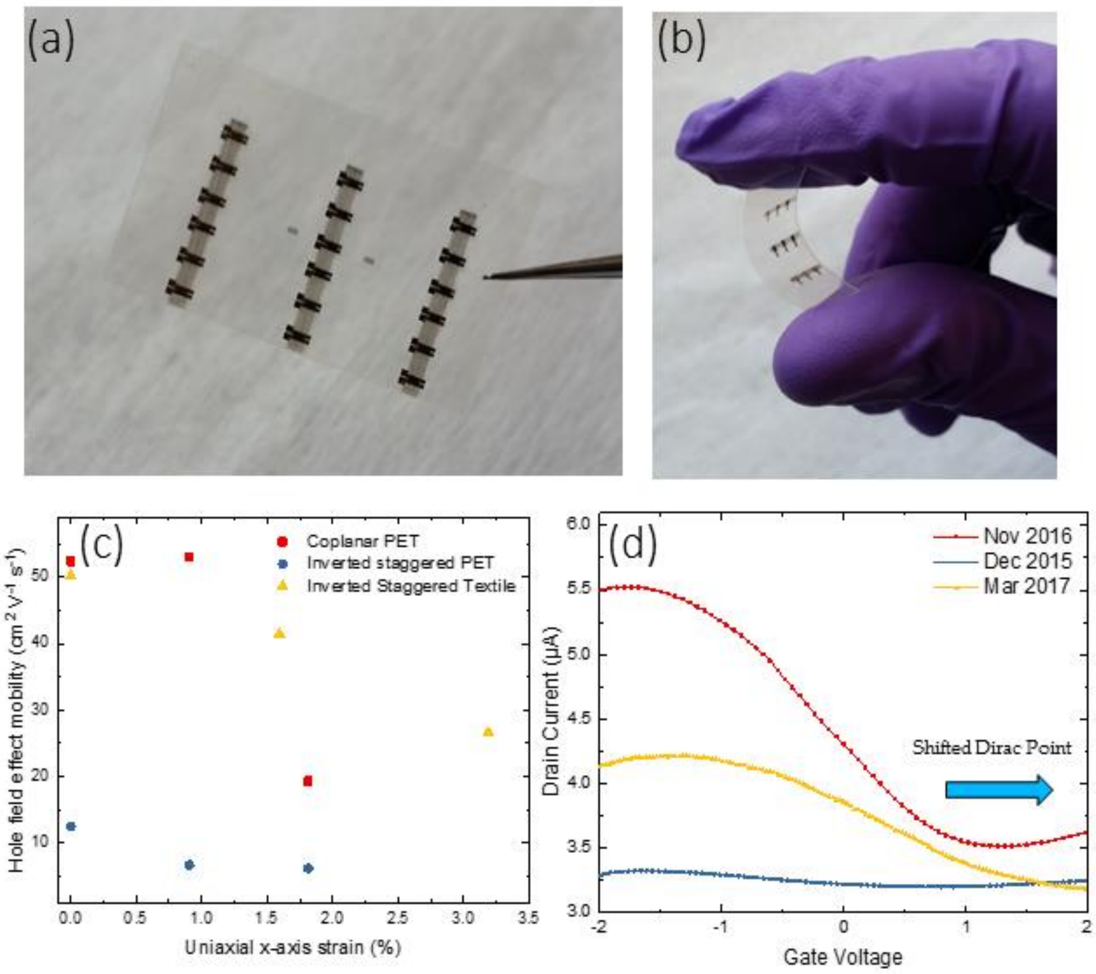

Supplementary Figure 4: Mechanical response and stability of the printed FET. (a) Image of coplanar TFTs array (3x6) on PET. (b) Image of the inverted staggered TFT array on PET. (c) Mobility as a function of uniaxial strain in the x-direction. (d) Evolution of the transfer characteristic over a 2 year period at V_{ds} = 50mV

Supplementary Note 2: Mobility as a function of uniaxial strain and Stability test

While the device characteristics were relatively unchanged we notice that the Dirac point slowly shifts over time (Supplementary figure 4d). It is likely oxygen is slowly diffusing though the h-BN layer and doping of the channel over time[2]. Moreover we notice that the on/off ratio increases slightly from 1.03 to 1.57, possibly due to an improvement in the graphene-metal contact resistance as a result of residual high boiling point solvents slowly evaporating from the silver contact[3].

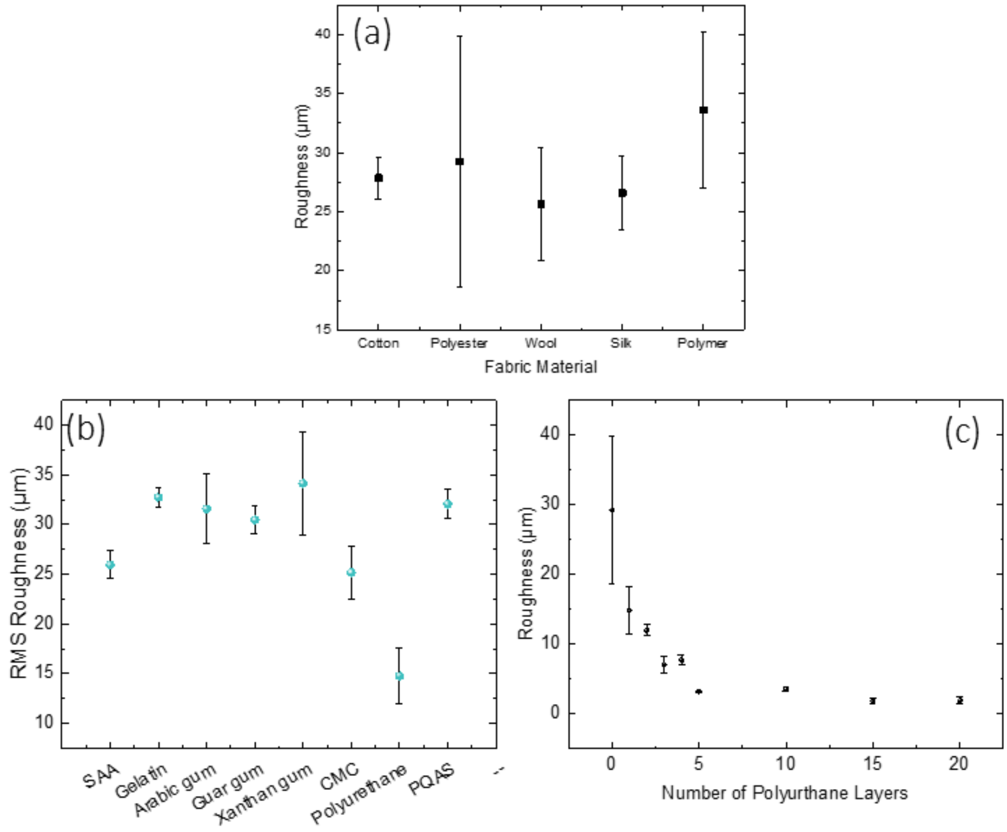

Supplementary Figure 5: Effect of textile planarization layers on the roughness. (a) "Roughness" (determined with a profilometer) of five different fabric materials. (b) Profilometry indicating R_q of the planarization layers. (c) Polyurthane planarization layer as a function of coating passes. All Error bars are calculated using standard deviation of mean.

Supplementary Note 3: Textile roughness and planarization layers

To determine a suitable planarization layer we rod coated (K202 RK coating machine) the polyester with eight different materials; sodium alga acid (SAA), gelatin, arabic gum, guar gum, xanthan gum, sodium carboxymethylcellulose (CMC), polyurethane, polymerizable quaternary ammonium salt

(PQAS) and measured R_q using a profilometer (DektakXT, Bruker) (Supplementary figure 5b). It is worth noting that the profilometer can only estimate R_q as the stylus tip is larger than an AFM tip so it will be unable to distinguish pinholes which are smaller than the tip diameter. After coating, the fabric is annealed at 60°C in an oven (Genlab) for 20min. We also investigated the effect of multi-stacked planarization layers by applying several coatings of polyurethane on polyester from 1 to 20 layers (Supplementary Figure 5c). We noticed a decrease of R_q as a function of the number of coating passes and after 20 layers (thickness ~12μm) we achieve an R_q decrease from 29±10 μm to 1.9 ± 0.5 μm.

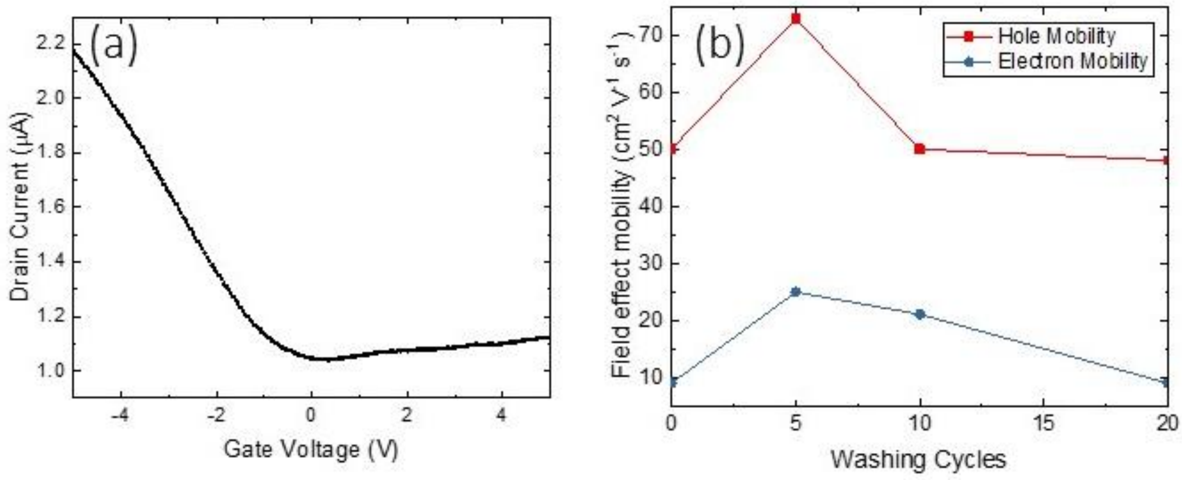

Supplementary Figure 6: Printed FET on textile fabric. (a) Transfer characteristic of the *t* ~100 nm graphene thickness textile TFT at Vds = 1 with L ≈ 80 μm, W ≈ 500 μm. (b) Field effect mobility as a function of washing cycles for the *t* ~200 nm graphene thickness textile TFT.

Supplementary Note 4: Textile Field effect transistor

Supplementary figure 6a plots the transfer characteristic of the graphene/h-BN FET heterostructure on textile with a graphene channel thickness of 100 nm. We observed ambipolar behaviour and obtain an average mobility μ_h = 8 ± 3 cm^2 V^{-1} s^{-1}, μ_e = 2 ± 1 cm^2 V^{-1} s^{-1} respectively, and on/off ratio of ~2.0 ± 0.1. These field effect mobilities are about one magnitude lower than μ_h and μ_e obtained on for the inverted staggered graphene/h-BN FET on PET, most likely due to the increase of the roughness of the h-BN layer from R_q = 68 nm on PET to R_q = 588 nm on textile which brings the graphene channel below the bulk conductivity regime.

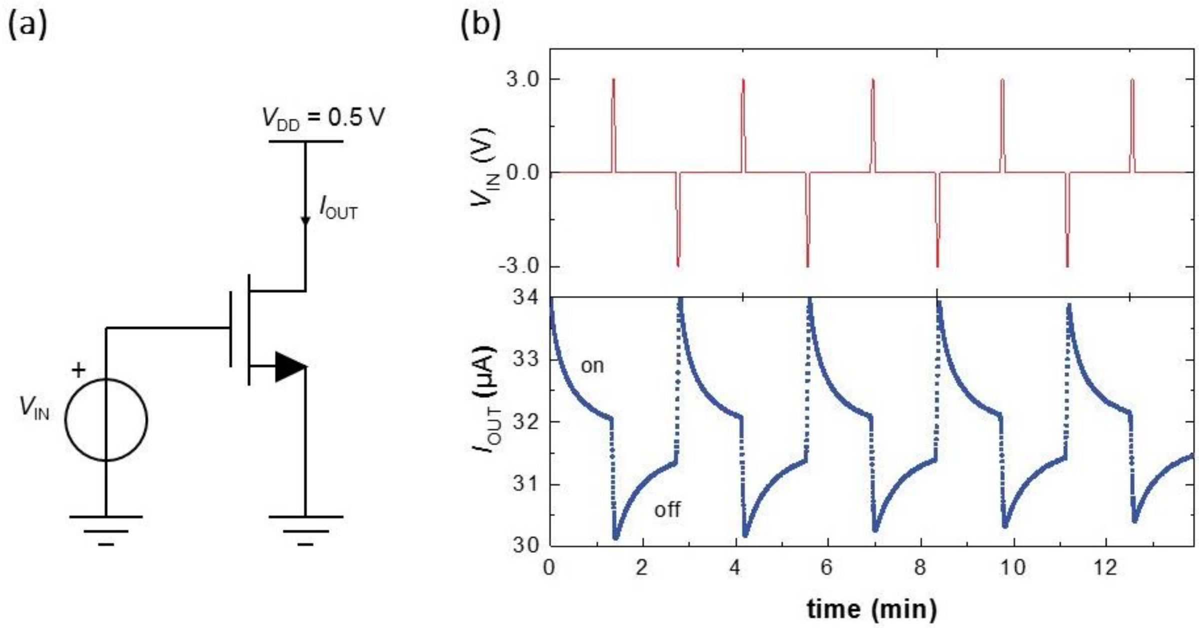

Supplementary Figure 7: Fully Inkjet Printed Reprogrammable Memory. (a) Schematic of memory cell at V_{DD} = 0.5 V which is tested under ambient conditions. (b) Memory effect in the graphene/h-BN FET coplanar heterostructure. Switching of the device is triggered by a signal with an amplitude of 3V. Digital waveforms are gained from the device indicating a rewritable memory which can be programmed into an on and off state.

Supplementary Note 5: Circuits based on all-printed 2d material heterstructures

All inkjet printed volatile memory cell: A graphene/h-BN FET coplanar heterostructure was used to create a rewriteable memory cell[4]. The static pulse response measurement of the device is presented in Supplementary Figure 7. Triggering was done using a pulse wave signal which a period of 3 minutes which ensures that a stable and well distinguished on and off states are reached. When the device is in the on state a positive gate voltage of 3V moved the operating point to the off state and remains in the off state even after the gate voltage is reset. Similarly a negative gate pulse of -3V moves the operating point to the on state which restores the off state of the memory. Therefore we predict that these device

could be first step towards fully printed volatile memory using 2d material with applications in RAM (random access memory) which is commonly used for storage in personal computers.

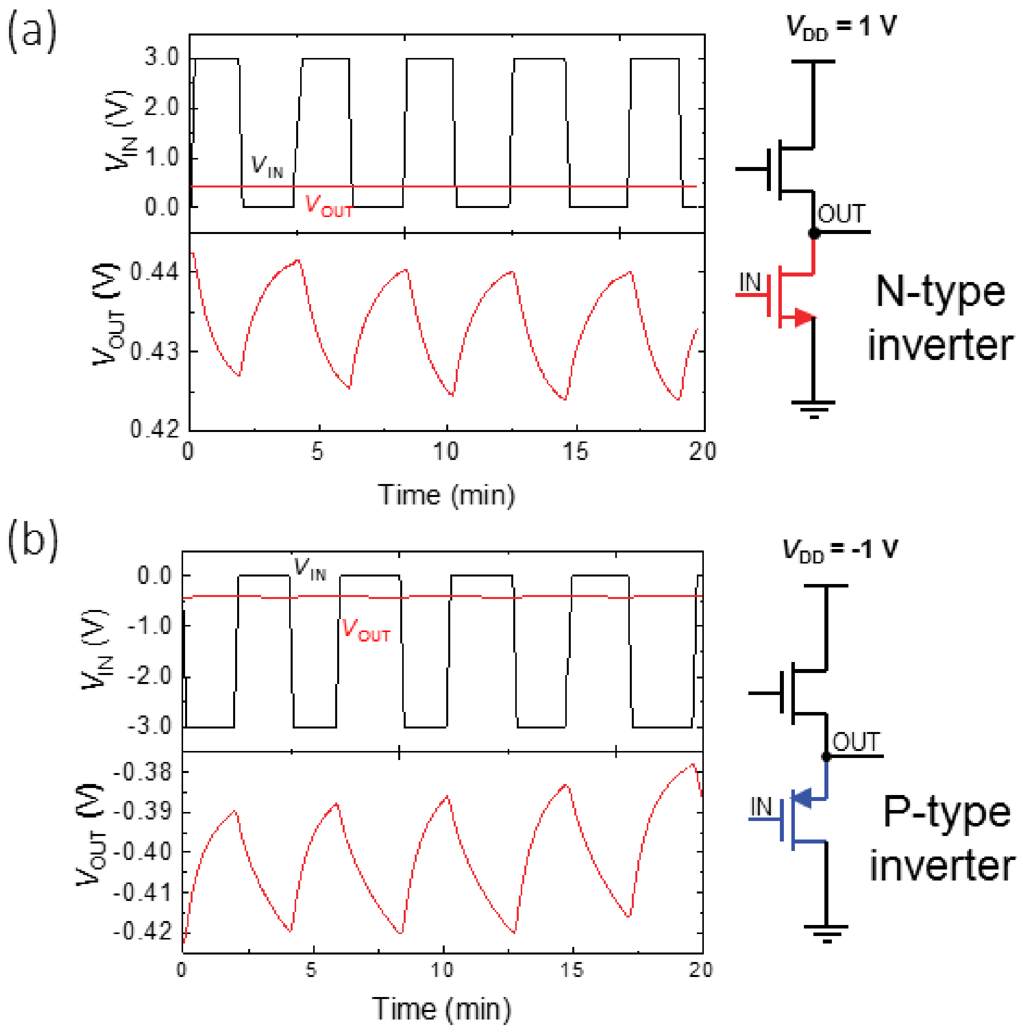

Supplementary Figure 8: Inkjet printed graphene/h-BN inverters. Digital waveforms for a n-type (a) and p-type (b) fully printed graphene inverters at V_{DD} = 1 V and V_{DD} = -1 V respectively. The devices were measured at ambient conditions.

All-printed n-type and p-type inverters: Graphene/h-BN FET coplanar heterostructures were used to create an n-type and p-type inverters. In this case, only one input is connected to the gate of the bottom FET, while the top FET is left floating (i.e. no bias) and used as a resistive load (pull-up resistor) of the bottom GFET. An n-type inverter is obtained if the input voltage (V_{in}) is above the Dirac voltage of the bottom transistor[5] (supplementary Fig. 8a). The p-type inverter is obtained if V_{in} falls below the Dirac voltage of the bottom GFET and a negative power supply is used (e.g. a buffer is obtained if a positive

power supply is used). In both cases, the output voltage swing is smaller than that of the complementary inverter shown in supplementary figures 6 b-e of the main text because the resistance of the top GFET is constant. All measurements were performed under ambient conditions.

Supplementary References

1. Kelly, A. G., Finn, D., Harvey, A., Hallam, T. & Coleman, J. N. All-printed capacitors from graphene-BN-graphene nanosheet heterostructures. *Appl. Phys. Lett.* **109,** (2016).
2. Lee, B. *et al.* Modification of electronic properties of graphene with self-assembled monolayers. *Nano Lett.* **10,** 2427–2432 (2010).
3. Xia, F., Farmer, D. B., Lin, Y. M. & Avouris, P. Graphene field-effect transistors with high on/off current ratio and large transport band gap at room temperature. *Nano Lett.* **10,** 715–718 (2010).
4. Stützel, E. U. *et al.* A graphene nanoribbon memory cell. *Small* **6,** 2822–2825 (2010).
5. Sordan, R., Traversi, F. & Russo, V. Logic gates with a single graphene transistor. *Appl. Phys. Lett.* **94,** (2009).
